# Integrin-Targeting Dye-Doped PEG-Shell/Silica-Core Nanoparticles Mimicking the Proapoptotic Smac/DIABLO Protein

**DOI:** 10.3390/nano10061211

**Published:** 2020-06-21

**Authors:** Rossella De Marco, Enrico Rampazzo, Junwei Zhao, Luca Prodi, Mayra Paolillo, Pierre Picchetti, Francesca Gallo, Natalia Calonghi, Luca Gentilucci

**Affiliations:** 1 Department of Agricultural, Food, Enviromental and Animal Sciences (DI4A), University of Udine, 33100 Udine, Italy; rossella.demarco@uniud.it; 2 Department of Chemistry “G. Ciamician”, University of Bologna, 40126 Bologna, Italy; enrico.rampazzo@unibo.it (E.R.); junwei.zhao2@unibo.it (J.Z.); luca.prodi@unibo.it (L.P.); francesca.gallo@hdpharma.com (F.G.); 3 Department of Drugs Sciences, University of Pavia, 27100 Pavia, Italy; mayra.paolillo@unipv.it; 4 Institut de Science et d’Ingénierie Supramoléculaires (ISIS), Université de Strasbourg, 67083 Strasbourg, France; picchetti@unistra.fr; 5 Department of Pharmacy and Biotechnology, University of Bologna, 40126 Bologna, Italy

**Keywords:** Smac/DIABLO, cancer, RGD, AVPI, IAP, confocal microscopy, silica nanoparticles, cellular uptake, drug delivery

## Abstract

Cancer cells demonstrate elevated expression levels of the inhibitor of apoptosis proteins (IAPs), contributing to tumor cell survival, disease progression, chemo-resistance, and poor prognosis. Smac/DIABLO is a mitochondrial protein that promotes apoptosis by neutralizing members of the IAP family. Herein, we describe the preparation and in vitro validation of a synthetic mimic of Smac/DIABLO, based on fluorescent polyethylene glycol (PEG)-coated silica-core nanoparticles (NPs) carrying a Smac/DIABLO-derived pro-apoptotic peptide and a tumor-homing integrin peptide ligand. At low μM concentration, the NPs showed significant toxicity towards A549, U373, and HeLa cancer cells and modest toxicity towards other integrin-expressing cells, correlated with integrin-mediated cell uptake and consequent highly increased levels of apoptotic activity, without perturbing cells not expressing the α5 integrin subunit.

## 1. Introduction

Apoptosis, or programmed cell death, is an essential process in the homeostasis of multicellular organisms. Apoptosis initiates through either the extrinsic death receptor pathway or the intrinsic mitochondrial signaling pathway, which both culminate with the activation of Cysteine ASPartic acid-specific proteASES (CASPASES), enzymes that degrade specific substrates implied in fundamental cellular processes. In mammals, caspase-3, -7 and -9 activity is regulated by the inhibitor of apoptosis proteins (IAPs) [1,2]. The mammalian IAP family includes eight members, all of which share the family-defining baculovirus IAP repeat (BIR) domain at the N-terminal end of the protein [3]. BIRs are protein-interacting modules with distinct binding properties, necessary for the anti-apoptotic activity [4,5,6].

A strict regulation of apoptosis is involved in many human diseases [7]. Tumorigenic cells exhibit significantly elevated expression levels of IAPs, resulting in the elusion of apoptosis, one of the defining hallmarks of cancer and an underlying cause of therapeutic resistance [8]. IAP-mediated caspase inhibition is depressed by the second mitochondria-derived activator (Smac)/direct inhibitor of apoptosis-binding protein with low pI (DIABLO), a mitochondrial protein that is translocated to the cytoplasm in apoptotic conditions [9]. Structural analysis proved that the N-terminal sequence of Smac/DIABLO is essential for its function in the interaction with the BIR domain of IAP [10]. As a consequence, peptides derived from the N-terminal sequence of Smac/DIABLO may represent attractive anticancer molecules.

In general, native peptides show too scarce stability and bioavailability to consent therapeutic or diagnostic applications [11,12]. To increase cellular uptake, the native seven-residue N-terminus of Smac/DIABLO (SmacN7) was bonded to a cell membrane-permeable octaArg peptide (R8). The resulting SmacN7-R8 was able to induce the apoptosis of human non-small lung cancer (NSCLC) cells H460 [13]. More recently, peptidomimetics [12] of the N-terminus of Smac/DIABLO with potentially higher stability and bioavailability, including retro-inverso [14], C-naphthyl substituted [15], and aza-peptides [16] have been designed and tested.

Nanoparticles (NPs) provide extraordinary opportunities as drug nanocarriers [17,18,19], due to their prolonged circulation time and both passive and active targeting abilities towards cancerous tissues/cells. The conjugation of peptides to NPs represents an effective approach to addressing the intrinsic drawbacks of the peptides, allowing the access to a variety of biomedical uses [20,21]. Specifically, this conjugation increases the circulating half-lives of the peptides in vivo, reducing the need for frequent administrations to sustain their efficacy [22]. As concerns the transport of Smac/DIABLO, Seneci et al. reported non-covalent and covalent superparamagnetic iron oxide NPs (SPIONs)–Smac/DIABLO mimetic nanoconjugates. Unfortunately, the nanoconjugates were almost inactive in assays against breast cancer MDA-MB-231 cells, ovarian carcinoma IGROV-1 cells, and cervical cancer HeLa cells [23]. Li et al. prepared a SmacN7-conjugated polymer containing the cell-penetrating R8 peptide and four hydrophobic tails. The Smac-conjugated polymer could self-assemble, giving NPs in the aqueous environment. At high concentrations (>10 μM), Smac–NPs elicited a measurable effect in MDA-MB-231 and H460 cells. The same NPs have been also used as a drug delivery system for doxorubicin (DOX) in combination therapy; DOX-loaded NPs exhibited higher cellular uptake and antitumor effect [24].

Finally, the tumor-targeting precision of drugs [25,26], NPs [27,28], polymers [29], biomaterials [30], or nanostructured materials [20,31,32] can be strongly improved by conjugation with peptide ligands addressing integrin receptors overexpressed by cancer cells [20,33,34]. The integrin family of cell adhesion receptors regulates a diverse array of cellular functions crucial to the initiation, progression, and metastatization of solid tumors, making them an appealing target for cancer therapy [35]. For this reason, Gennari et al. connected a cyclo Arg–Gly–Asp (cRGD) ligand of the integrins αvβ3 and αvβ5 to mimetics of Smac/DIABLO. In vitro, the conjugates showed moderate synergistic/enhanced cytotoxic effects towards IGROV-1 cells [36].

In this context, we designed nanosystems mimicking the Smac/DIABLO protein, based on inorganic fluorescent NPs coated with a biocompatible organic shell, functionalized with a Smac/DIABLO-derived peptide and/or a tumor-homing RGD integrin ligand peptide. We opted for micellar NPs composed of the tri-block surfactant copolymer Pluronic^®^ F127 (PF127) (polyethylene glycol-polypropyleneoxide-polyethylene glycol, PEG_100_–PPO_65_–PEG_100_) and a dye-doped silica core. The PEG–PPO–PEG block copolymers alone can form micelles in aqueous media with a hydrophilic core, which can be used to non-covalently encapsulate hydrophobic dyes with minimal leakage [37,38]. Micelles are valuable systems for application in the field of imaging and drug delivery, since they are self-organized systems, and the encapsulation of dyes and drugs in these systems is straightforward from the preparation point of view. However, they are dynamic systems, and their stability is strongly influenced by their local concentration, pH, and eventually by the presence of other species like apolar molecules or proteins [21,38].

On the other hand, silica NPs as tools to develop targeting probes have several advantages over other nanomaterial and self-organized systems [39]. Indeed, silica is photophysically inert, is an intrinsically non-toxic material, and there are many synthetic approaches available to tune these nanosystems in terms of size and functionalization. The luminescence emission of these systems depends on the doping dye, so that a large variety of emission properties can be achieved by just choosing the right doping dye(s). The inclusion of dye molecules in a rigid matrix like silica often increases the quantum yield of the dyes and also their photostability, because of the rigidification of dye structure and the protection towards quenching molecules present in the environment [39]. These last two features are of prominent importance to univocally assign the recorded fluorescent signal to the presence of the NPs and to control the local concentration of the cytotoxic compound during the recognition event toward the targeted receptor.

For the purpose of peptide conjugation, NPs were prepared from a mixture of PF127 and its diazide derivative PF127-(N_3_)_2_. After synthesis of the NPs and characterization, the azide terminations of the outer shell were exploited to covalently bind a Smac/DIABLO-derived peptide and/or a tumor-homing integrin ligand peptide. Then, the cytotoxicity, pro-apoptotic efficacy, and cellular uptake were determined for these peptide–NPs in diverse cells. Particularly, the role of integrin-mediated cell uptake was investigated by confocal microscopy.

## 2. Materials and Methods

### 2.1. Chemistry

#### 2.1.1. General Methods

Standard chemicals, including protected amino acids, were purchased from commercial sources and used without further purification. Peptide purity was assessed by analytical RP HPLC performed on an 1100 series apparatus Agilent Technologies, Milan, Italy, using an XSelect Peptide CSH C18 column (Waters, Milford, MA, USA), 4.6 mm × 100 mm, 130 Å, 3.5 μm. MS (ESI) analysis was performed using an MS single quadrupole HP 1100 MSD detector (Agilent Technologies, Milan, Italy). The synthetic procedures by MW irradiation were performed with a Microwave Labstation for Synthesis (Micro-SYNTH, Bergamo, BG, IT) equipped with a built-in ATC-FO advanced fiber-optic automatic temperature control. Peptides isolation was performed by preparative RP HPLC performed on an 1100 series apparatus (Agilent), using an XSelect Peptide CSH C18 OBD column (Waters) 19 mm × 150 mm, 130 Å, 5 µm. The molecular weights of the purified peptides were verified by electrospray ionization (ESI)–mass spectrometry (MS) using an MS single quadrupole HP 1100 MSD detector (Agilent). Fluorescence measurements were performed with an LS-55 Fluorescence Spectrometer (Perkin Elmer, Milan, Italy). DLS measurements were performed with a Zetasizer Nano ZS (Malvern Panalytical, Malvern, UK). For full details, please see the Supporting Information.

#### 2.1.2. c[Arg–Gly–Asp–D-Phe–Lys(hex–5–ynamide)] (cRGD–alkyne)

The linear precursor was obtained as reported in the literature [40]. In brief, H–Asp(OtBu)–D-Phe–Lys(Cbz)–Arg(Mtr)–Gly–OH was prepared by solid-phase peptide synthesis (SPPS) on a 2-chlorotritil chloride resin, by coupling fluorenmethyloxycarbonyl (Fmoc)-protected amino acids with 1-hydroxybenzotriazole/N,N,N′,N′-tetramethyl-O-(1H-benzotriazol-1-yl)uronium hexafluorophosphate/N,N-diisopropylethylamine (HOBt/HBTU/DIPEA) under microwave (MW) irradiation according to a recently optimized procedure [41]. Fmoc was removed with piperidine/dimethylformamide (DMF) under MW irradiation. After cleavage from the resin with AcOH/2,2,2-trifluoroethanol/dichloromethane (DCM), the crude peptide was submitted to cyclization in the presence of diphenyl phosphoryl azide/NaHCO_3_ in DMF under pseudo high-dilution conditions [42]. The carbobenzyloxy (Cbz)-protecting group at Lys was removed by catalytic hydrogenation, then the εNH_2_ amine was derivatized with 5-hexynoic acid in DMF/DCM with HOBt/TBTU/DIPEA under MW, giving c[Arg(Mtr)–Gly-Asp(OtBu)–D-Phe–Lys(hex-5-ynamide)]. Finally, the 4-methoxy-2,3,6-trimethylbenzenesulphonyl (Mtr) and tert-butyl (tBu) side-chain-protecting groups were removed with trifluoroacetic acid (TFA) and a cocktail of scavengers. Full details are given in the Supporting Information.

#### 2.1.3. H–Ala–Val–Pro–Ile–Gly–pent-4-yn-1-amine (AVPI–alkyne)

The precursor Boc–Ala–Val–Pro–Ile–Gly–OH was prepared by SPPS and cleavage using the same protocol as described above; all details are given in the Supporting Information. Briefly, the crude peptide was coupled to 4-pentyn-1-amine with HOBt/TBTU/DIPEA in DMF/DCM, under MW irradiation, and the tert-butyloxycarbonyl (Boc) was removed with TFA.

#### 2.1.4. PEG-Shell/Silica-Core Azide–NPs (NP–N_3_)

The dimesylate derivative of BASF Pluronic^®^F127 (PF127), obtained in turn by the treatment of PF127 surfactant with trimethylamine/methanesulfonyl chloride, was reacted with NaN_3_. The resulting PF127–(N_3_)_2_ (20 mg) was mixed with PF127 (200 mg) and RhB-TES (4.0 mg) in DCM, followed by tetraethyl orthosilicate (TEOS, 350 μL) and trimethylchlorosilane (40 μL) in the presence of AcOH/NaCl. The obtained NPs were purified by dialysis and diluted with water to the final concentration of 29 μM [43]. For full details, please see the Supporting Information.

#### 2.1.5. NP–N_3_ Functionalization with cRGD–alkyne and/or AVPI–alkyne

The NP–N_3_ (500 μL, 30 μM) were dispersed in 1 mL of water and Tris-buffer pH 8 (1.5 mL, 200 mM) and treated with CuSO4 (6 μL, 2 mM), sodium 4,4′-(1,10-phenanthroline-4,7-diyl)dibenzenesulfonate (12 μL, 2 mM), peptide–alkyne (40 equiv. respect to NPs), and polished copper wire. After 3 days at rt, the NPs were purified by size-exclusion chromatography on Sephadex^®^ G-25 gel using bidistilled water and finally diluted to the final NP concentration of 3 μM [43]. The full description is given in the Supporting Information.

### 2.2. Biological Methods

#### 2.2.1. Cells and Culture Conditions

Human umbilical vein endothelial cells (Huvec), adenocarcinoma human alveolar basal epithelial cells (A549), human glioblastoma (U373), and human fibroblasts were obtained from Thermofisher Scientific, Waltham, MA, USA. The human cervical carcinoma (HeLa) cells and human colon cancer (HT29) cells were obtained from ATCC. DMEM, trypsin, PBS, Gly, and BSA 1% were purchased from Merck Co Ltd., Serono, Italy. Mouse anti-α-tubulin primary antibody was purchased from BioLegend, San Diego, CA. Anti-mouse fluorescein isothiocyanate (FITC)-conjugated secondary antibody and Hoechst33342 were purchased from ThermoFisher. The MTS assay CellTiter 96^®^ AQueous One Solution Cell Proliferation Assay and Caspase-Glo^®^ 9 assay were purchased from Promega, Italy. A Synergy HT microplate reader Biotek, Milan, Italy, was used.

The cells were grown in RPMI 1640 medium (Labtek Eurobio, Milan, Italy), supplemented with 10% FCS (Euroclone, Milan, Italy) and 2 mM L-glutamine (Sigma-Aldrich, Milan, Italy), at 37 °C and in a 5% CO_2_ atmosphere. The cells were seeded at 20 × 10^4^ cells/cm^2^ in plastic wells (Orange Scientific, Brainel’Alleud, Belgium). The cells were detached by a trypsin–EDTA solution (0.115 w/v % trypsin and 0.02 w/v % EDTA) (Sigma-Aldrich, Milan, Italy) and then rinsed and re-suspended in the corresponding medium.

#### 2.2.2. Cell Viability Assays

The cytotoxicity of peptide–NPs was evaluated using the cell viability 3-(4,5-dimethylthiazol-2-yl)-5-(3-carboxymethoxyphenyl)-2-(4-sulfophenyl)-2H-tetrazolium (MTS) assay, according to the manufacturer’s instructions. Cells were seeded (1.5 × 10^4^ cells/well) and cultured for 48 h. The primary growth medium was replaced by fresh medium, containing NPs at the concentrations of 0.1, 1.0, 3.0 µM. After 48 h, PBS (100 μL) was supplemented with the MTS solution (20 μL/well), incubated for 2 h, and then the absorbance was recorded at 570 nm with a 96-well plate reader. Data were analyzed by Prism software (GraphPad) and expressed as % of controls (untreated cells).

#### 2.2.3. Apoptosis

We incubated 1.0 × 10^4^ cells/well with the peptides–NPs at the concentration of 1 µM for 6 h. The apoptotic process onset was evaluated by the Caspase-Glo^®^ 9 assay (Promega), according to the manufacturer’s instructions. After 30 min, the luminescence was measured using a Synergy HT microplate reader (Biotek).

#### 2.2.4. Cell Internalization

HT29 and HeLa cells were grown on sterile glass coverslips for 48 h and then treated with 1 µM peptide–NPs for 1 h. The cells were washed (3×) with PBS and fixed in 500 μL of 3% paraformaldehyde. The glass slides were washed twice with 1 mL of PBS–Gly 0.1 M and washed twice again with 1 mL of PBS–BSA 1%. The samples were first incubated with mouse anti-α-tubulin primary antibody for 1 h in agitation at rt. The samples were washed again twice with 1 mL of PBS–BSA 1% and then incubated with anti-mouse FITC-conjugated secondary antibody for 1 h at rt. Finally, the specimens were embedded in Mowiol and analyzed by confocal microscopy. Confocal images were obtained with a C1s confocal laser-scanning microscope equipped with a PlanApo, 60X or 40X, oil immersion lens (Nikon, Tokyo, Japan). The visualization and quantification of cells that internalized the rhodamine B (RhB)–NPs were performed using ImageJ (NIH, Bethesda, MD, USA).

#### 2.2.5. Competition Experiments

HeLa cells were seeded on sterile glass coverslips for 48 h. Then, the cells were first pre-exposed to IgG isotype or anti-CD49e antibody for 1 h and then incubated with 1 µM peptide–NP for 1 h. The cells were stained with Hoechst33342, and the specimens were analyzed by confocal microscopy as described above. The number of cells, counterstained with Hoechst 33342, showing intracellular red fluorescence was expressed as % of the total cells.

## 3. Results

### 3.1. Chemistry

**Figure 1:**
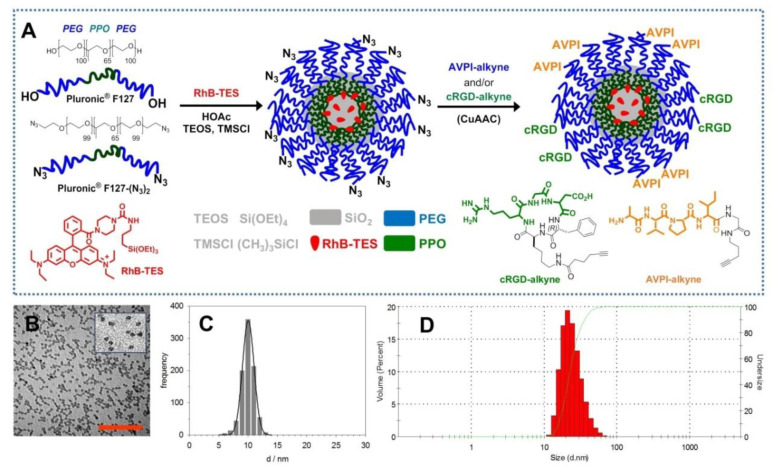
(**A**) Molecular components involved in the synthesis of nanoparticles (NPs)–N_3_ and functionalization scheme with the peptides H–Ala–Val–Pro–Ile (AVPI)–alkyne and/or cyclo Arg–Gly–Asp (cRGD)–alkyne for the preparation of AVPI–NPs, cRGD–NPs, and AVPI/cRGD–NPs. Morphological characterization of NPs–N_3_: (**B**) TEM images of NP–N_3_ (scale bar = 100 nm) and (**C**) TEM distribution of the diameters (nm). (**D**) Hydrodynamic diameters distribution by volume of NP–N_3_ determined by DLS (water, 25 °C). PEG, polyethylene glycol, PPO, polypropyleneoxide, RhB, rhodamine B, TEOS, tetraethyl orthosilicate, TMSCl, trimethylchlorosilane.

Monodispersed fluorescent silica-core/PEG-shell NPs functionalized with azide moieties and incorporating the dye rhodamine B triethoxysilane (RhB-TES) [44] (Figure 1A) were expediently obtained using a direct micelle-assisted method [45]. These nanostructures were formed by the condensation of the silica precursor TEOS in an aqueous acid environment in the presence of co-aggregates composed by a 10:1 mixture of the tri-block surfactant copolymer PF127 and its diazide derivative PF127–(N_3_)_2_ [43]. The condensation of RhB-TES within the silica core of the NP conferred the desired fluorescent properties to this nanosystem, preventing also the leaking of the fluorophore in the external environment. Transmission electron microscopy (TEM) images showed a silica core diameter *dc* = (10 ± 2) nm, while the hydrodynamic diameter measured by dynamic light scattering (DLS) was dH = 22 ± 1 nm (PDI = 0.10) (Figure 1B–D), confirming the core/shell type architecture of the resulting NPs–N_3_.

The NPs–N_3_ were derivatized by copper (I)-catalyzed azide–alkyne cycloaddition (CuAAC) with the peptide–alkyne AVPI-alkyne, containing the pro-apoptotic Smac/DIABLO-derived sequence AVPI [10], the integrin-targeting cyclopeptide–alkyne cRGD-alkyne [46], or a 1:1 mixture of both. These reactions gave AVPI–NPs, cRGD–NPs, and AVPI/cRGD–NPs, respectively. DLS indicated the effectiveness of the conjugation reaction, since the volume distribution after the conjugation reaction increased to 28 nm, compared to that of the pristine NP–N_3_ (Figures S1–S3 and Table S1).

By adapting reported procedures [47], the number of NP-bonded AVPI molecules was estimated by fluorimetric quantitation with fluorescamine (an amine-reactive fluorogenic tracer), against a standard calibration curve obtained with PEG–amine/fluorescamine (λex 390 nm, λem 480 nm). An aliquot of the AVPI–NP suspension (25 μL, 29 mM) was treated with fluorescamine, and the relative fluorescence intensity measured allowed to estimate 7.8 ± 1 peptides/NP. Alternatively, NPs functionalization was appraised by the fluorimetric quantitation of the dansyl group after CuAAC reaction with dansyl–AVPI–alkyne (Supporting Information), against a calibration curve obtained with unconjugated dansyl–AVPI–alkyne (λex 340 nm, λem 477 nm). Consistent with the fluorescamine method, this test gave 9.3 ± 1 dansyl–AVPI/NP and gave us the possibility to measure the amount of dansyl–AVPI bounded to a sample obtained by CuAAC reaction with a 1:1 mixture of dansyl–AVPI–alkyne and cRGD–alkyne. For this sample, an average number of 4.8 ± 1 dansyl–AVPI/NP was determined, indicating by the difference that cRGD-alkyne reacted circa to the same extent.

### 3.2. Cytotoxicity of Peptide–NPs

**Figure 2:**
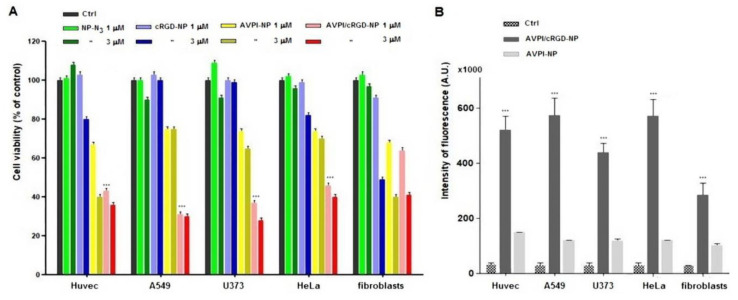
(**A**) Cell viability of peptide–NPs as % of control cells. Bars represent the mean ± SD from two independent experiments (n = 2), each performed in triple. (**B**) Caspase-9 levels after 6 h of incubation with either 1 µM AVPI/cRGD–NPs or AVPI–NPs. Bars indicate the increase in activity of the treated cells compared to the control. Data are reported as mean ± SD from at least three independent experiments. *** *p* < 0.001.

The in vitro cell growth inhibitory efficacy was determined for the NPs and the unconjugated AVPI peptide, by incubating A549, U-373, HeLa, Huvec, and fibroblast cells with increasing concentrations of the compounds (0.1, 1.0, 3.0 μM) for 48 h. Cell viability is reported in Figure 2A; in general, all the combinations tested were ineffective at the concentration of 0.1 μM; therefore, these data were omitted. As expected, the simple peptide AVPI did not show any toxicity (data not shown), plausibly due to poor-to-null intracellular uptake [24]. The NP–N_3_ appeared well tolerated, since no detectable decrease in cell viability was observed after 48 h.

As for the peptide–NPs, 1 μM cRGD–NPs showed very little toxicity, and modest toxicity when the concentration was increased to 3 μM. At the concentration of 1 μM, AVPI–NPs induced a decrease of viability of about 25% in A549, U373, and HeLa cells, and of 33% in Huvec and fibroblast cells. At the concentration of 3 μM, AVPI–NPs showed much higher toxicity against Huvec and fibroblasts, reducing their viability by 60%, while the effect was lower in U373, A549, and HeLa cells, whose vitality was decrased by 37% and 30%, respectively. In contrast, 1 μM AVPI/cRGD–NPs significantly inhibited the proliferation of A549, U373, HeLa, and Huvec cells of about 60% and showed a comparatively lower effect towards fibroblasts. Increasing the concentration of AVPI/cRGD–NPs to 3 μM led in general to higher toxicity, whit the exclusion of A549 cells for which the toxicity remained the same.

### 3.3. Caspase-9 Activity

The activity of caspase-9 [9] was assayed by a fluorimetric method in A549, U373, HeLa, Huvec, and fibroblast cells treated with 1 µM peptide–NPs for 6 h (Figure 2B). The AVPI–NPs gave a moderate but well measurable increase of activity, about four-fold, as compared to untreated control cells. On the other hand, AVPI/cRGD–NPs showed a >40-fold increase towards A549 and HeLa cells, Huvec, U373, and a comparatively lower 10-fold increase in activity in fibroblasts.

### 3.4. Cellular Uptake of Peptide–NPs

**Figure 3:**
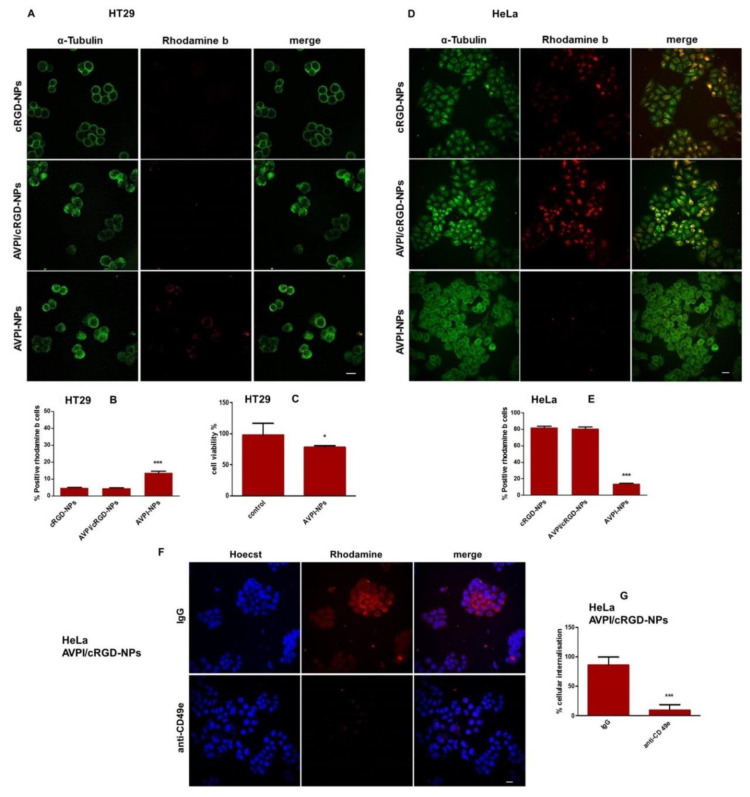
Fluorescence microscopy of cells after 1 h of treatment with fluorescent peptide–RhB–NPs (red), counterstained with anti α-tubulin antibody (green) to visualize the cytoskeleton. (**A**) HT29 cells. Photographs were taken at 60× magnification, bar = 20 µm. (**B**) RhB-positive HT29 cells %, error bars represent SD (n = 15 imaging fields), *** *p* < 0.001. (**C**) HT29 cell viability % in the presence of AVPI–NPs; data represent mean ± SD (n = 6). * *p* < 0.05. (**D**) HeLa cells. Photographs were taken at 40× magnification. Bar 20 µm. (**E**) RhB-positive HeLa cells %, error bars represent SD (n = 15 imaging fields), *** *p* < 0.001. (**F**) HeLa cells were treated or not with an anti-CD49e antibody or control IgG for 1 h, then incubated whit fluorescent AVPI/cRGD–NPs (red) and counterstained with Hoechst33342 dye (blue) to visualize the nuclei. Photographs were taken at 40× magnification. (**G**) HeLa cells internalization %, error bars represent SD (n = 15 imaging fields), bar = 20 µm, *** *p* < 0.001.

The internalization of the fluorescent peptide–NPs was observed by confocal microscopy in HeLa (α5 subunit-positive) [48] and in HT29 (α5 subunit-negative) cells [49,50]. In control HT29 cells, the internalization of AVPI–NPs was modest, and that of cRGD–NPs and AVPI/cRGD–NPs was very poor, as shown by the low fluorescent signal in the cytoplasm (Figure 3A,B). By contrast, HeLa cells exposed to cRGD–NPs and AVPI/cRGD–NPs, but not AVPI–NPs, gave a similar, very high number of fluorescence-positive cells (Figure 3D,E). The scarce internalization of AVPI–NPs was consistent with the modest decrease of viability of HT29 (about 20%, Figure 3C) and HeLa (approx. 25%, Figure 2A) cells.

The merge of the images of HeLa treated with cRGD–NPs or AVPI/cRGD–NPs showed some cells colored in yellow/orange, but this observation was not indicative of colocalization between RhB and α-tubulin. These interactions were quantified by analyzing the correlation and/or the overlap between images, using the Pearson’s and Manders’ coefficients, respectively.

Finally, the cells were pre-incubated with anti-CD49e antibody or mouse immunoglobulin G (IgG) antibody before exposure to AVPI/cRGD–NPs (Figure 3F). Microscopic observations showed that, in the presence of the antibody, the internalization was considerably reduced, as compared to cells incubated in the presence of control IgG (Figure 3G).

## 4. Discussion

The aim of our study was to develop fluorescent probes with sufficient brightness, able to selectively sustain a long-term interaction with cellular receptors in dilute conditions. High brightness, low in vivo toxicity, and ease of functionalization with pharmacologically active biomolecules make fluorescent silica-based NPs attractive platforms for diagnostic and theranostic applications in cancer [39]. Hence, we prepared dye-doped silica NPs surrounded by an outer shell of the biocompatible polymer PEG, which is expected to increase NP dispersion in physiological conditions (Figure 1A) and to oppose the uptake by the reticuloendothelial system [51,52]. The covalent inclusion of the RhB derivative was adopted to prevent dye leaking in the external environment, a behavior that can affect the signal-to-noise ratio during optical imaging experiments. The influence of “minimal leakage” of fluorescent dyes from NPs or nanosystems on the overall fluorescent signal recorded during real experiments with cells is often a very difficult variable to quantify. For this reason, we preferred to circumvent this problem by the covalent linking of the dye to the NP silica core.

To avoid the leakage of the cytotoxic and targeting compounds, the azide termini of PEG were exploited for the covalent functionalization with peptide–alkyne partners. We designed the sequence AVPI–alkyne: the AVPI N-terminal tetrapeptide of Smac/DIABLO maintained a binding affinity for IAP BIR of 0.5 μM [10], while Gly and the C5 amine served as spacers. The cRGD–alkyne sequence was designed on the basis of the well-known Kessler’s α5β1 integrin ligand c[RGDfK] [46]. It is well known that simple peptides, such as the AVPI and RGD sequences, delivered to the body are subject to enzymatic degradation and are poorly permeable through biological membranes. Nevertheless, inorganic NP carriers can support the transport of peptides by protecting them from environmental conditions while maintaining their stability [20,22].

The stability of the NPs was previously tested under different pseudo physiological and in vivo experimental situations. Pluronic^®^F127/silica-core/PEG-shell NPs, doped with RhB and/or polymethine cyanine dye, demonstrated outstanding stability in the presence of phosphate-buffered saline and bovine serum albumin (PBS/BSA) [53]. These NPs were tested in small animals for in vivo total-body imaging and intravital 3D imaging, giving well detectable signals for hours after injection. The same silica-core/PEG-shell NPs, doped with cyanine 7 dye, were subcutaneously injected in animals, and the in vivo fluorescence signal in the right axillary lymph node was detected for at least 8 h [54].

The cytotoxicity of the resulting peptide–NPs was evaluated in A549, U-373, HeLa, Huvec, and fibroblast cells. Compared to AVPI–NPs, the dual functionalized AVPI/cRGD–NPs showed much higher toxicity towards the cancer cells and reduced toxicity towards fibroblasts. Indeed, the AVPI/cRGD–NPs inhibited the proliferation of A549, U373, HeLa, and Huvec cells of about 60% already at the concentration of 1 μM, a significantly higher efficacy as compared to that of the previously reported Smac/DIABLO–NPs [23,24]. To understand whether the peptide–NPs restored apoptotic cancer cell death, the activity of caspase-9 was measured. While AVPI–NPs gave a moderate increase of caspase activity, AVPI/cRGD–NPs showed a >40-fold increase in A549, HeLa, Huvec, and U373 cells and a comparatively lower effect in fibroblasts.

The much higher apoptotic effect of AVPI/cRGD–NPs over AVPI–NPs towards cancer cells appeared clearly correlated to integrin-mediated cellular uptake. Internalization of peptide–NPs was observed in HeLa (α5 subunit-positive) and in control HT29 (α5 subunit-negative) cells. cRGD–NPs and AVPI/cRGD–NPs showed much higher internalization in HeLa cells (Figure 3D,E) than AVPI–NPs, likely mediated by the interaction between the RGD peptides and the integrin receptors. This observation was supported by the almost negligible uptake of both RGD–NPs and AVPI/cRGD–NPs in the HT29 cell line not expressing the α5 subunit (Figure 3A). To confirm that the uptake of AVPI/cRGD NPs was integrin-mediated, exclusion studies were carried out by incubating the cells with anti-CD49e antibody or mouse IgG antibody before exposure to AVPI/cRGD–NPs. Microscopic observations showed that, in the presence of anti-CD49e, the internalization was strongly reduced, as compared to cells incubated in the presence of the control IgG (Figure 3F).

## 5. Conclusions

In this paper, we described synthetic NPs mimicking the proapoptotic protein Smac/DIABLO, constituted by a fluorescent silica core doped with RhB, coated with a PEG shell, and carrying the AVPI peptide and/or a tumor-homing cRGD peptide. The bifunctional AVPI/RGD–NPs showed superior toxicity towards cancer cells, correlated to increased levels of caspase activity, plausibly due to efficient integrin-mediated transport into the cells, as shown by confocal microscopy, and modest toxicity towards cells not expressing the α5 integrin subunit. In perspective, these Smac/DIABLO-mimetic nanosystems can find applications in the treatment of cancer and, thanks to the combination with the fluorescent dye, they can provide new insight into integrin-mediated internalization.

## Supplementary Materials

The following are available online at https://www.mdpi.com/2079-4991/10/6/1211/s1, Experimental details (peptides, nanoparticles); Table S1: NPs hydrodynamic diameters; Figure S1: DLS hydrodynamic size and TEM of AVPI–NPs; Figure S2: DLS hydrodynamic size and TEM of cRGD–NPs; Figure S3: DLS hydrodynamic size and TEM of AVPI/cRGD–NPs; Figure S4: Peptide/NP loading, fluorescamine; Figure S5: Dansyl–AVPI/NP loading.
