# Supplementary material for: Integrin-Targeting Dye-Doped PEG-Shell/Silica-Core Nanoparticles Mimicking the Proapoptotic Smac/DIABLO Protein
Source: Nanomaterials (Basel). 2020 Jun 21;10(6):1211. doi: 10.3390/nano10061211 (PMC7353088; doi:10.3390/nano10061211)
Supplement: Supplementary file 1 [file nanomaterials-10-01211-s001.pdf]

nanomaterials

Supplementary Material

Integrin-Targeting Dye-Doped PEG-Shell/Silica-Core Nanoparticles Mimicking the Proapoptotic Smac/DIABLO Protein

Rossella De Marco [1,†], Enrico Rampazzo [2,†], Junwei Zhao [2], Luca Prodi [2], Mayra Paolillo [3], Pierre Picchetti [4], Francesca Gallo [2], Natalia Calonghi [5,*] and Luca Gentilucci [2,*]

[1] Department of Agricultural, Food, Enviromental and Animal Sciences (DI4A), University of Udine, 33100 Udine, Italy; rossella.demarco@uniud.it
[2] Department of Chemistry "G. Ciamician", University of Bologna, 40126 Bologna, Italy; enrico.rampazzo@unibo.it (E.R.); junwei.zhao2@unibo.it (J.Z.); luca.prodi@unibo.it (L.P.); francesca.gallo@hdpharma.com (F.G.)
[3] Department of Drugs Sciences, University of Pavia, 27100 Pavia, Italy; mayra.paolillo@unipv.it
[4] Institut de Science et d'Ingénierie Supramoléculaires (ISIS), Université de Strasbourg, 67083 Strasbourg, France; picchetti@unistra.frin
[5] Department of Pharmacy and Biotechnology, University of Bologna, 40126 Bologna, Italy
* Correspondence: natalia.calonghi@unibo.it (N.C.); luca.gentilucci@unibo.it (L.G.); Tel.: +39-051-209-1231 (N.C.); Tel.: +39-051-209-9570 (L.G.); Fax: +39-051-209-9456 (L.G.)
† These authors contributed equally.

General methods

Standard chemicals, including protected amino acids, were purchased from commercial sources and used without further purification. Peptide purity was assessed by analytical RP HPLC performed on a 1100 series apparatus (Agilent). Method A: RP XSelect Peptide CSH C18 column (Waters), 4.6 mm × 100 mm, pore size 130 Å, particle size 3.5 µm; mobile phase: from 9:1 H_2O/0.1% TFA//CH_3CN/0.1% TFA to 2:8 H_2O/0.1% TFA//CH_3CN/0.1% TFA, in 20 min at a flow rate of 1.0 mL/min, followed by 10 min at the same composition. Method B: for fully protected peptide intermediates, the same chromatographic system was used with the exclusion of TFA in the mobile phase. Peptide isolation was performed by preparative RP HPLC performed on an 1100 series apparatus (Agilent), using a XSelect Peptide CSH C18 OBD column (Waters), 130 Å, 5 µm, 19 mm × 150 mm; mobile phase: from 7:3 H_2O/0.1% TFA//CH_3CN/0.08% TFA to 2:8 H2O/0.1% TFA//CH_3CN/0.08% TFA, in 10 min at a flow rate of 10 mL/min. MS (ESI) analysis was performed using an MS single quadrupole HP 1100 MSD detector (Agilent), with a drying gas flow of 12.5 L/min, nebulizer pressure 30 psi, drying gas temp. 350 °C, capillary voltage 4500 (+) and 4000 (−), scan 50–2600 amu. The synthetic procedures by MW irradiation were performed with a Microwave Labstation for Synthesis (Micro-SYNTH) equipped with a built-in ATC-FO advanced fiber fiber-optic automatic temperature control. Fluorescence measurements were performed with an LS-55 Fluorescence Spectrometer (Perkin Elmer) and quartz cuvettes, optical path length 1 cm. DLS measurements were performed with a Zetasizer Nano ZS (Malvern), He-Ne laser 633 nm, Max 4 mW, using polystyrene cuvettes (optical path length 1 cm). Confocal images were obtained with a C1s confocal laser-scanning microscope (Nikon), equipped with a PlanApo 60× or 40×, oil immersion lens (Nikon).

Solid-phase synthesis of H–Asp(OtBu)–D-Phe–Lys(Cbz)–Arg(Mtr)–Gly–OH

H–Gly-preloaded 2-chlorotrityl resin (0.5 g, Gly loading 1.1 mmol/g resins) was swollen in DCM (5 mL) for 10 min before use. Fmoc-amino acid (2 equiv.) carrying orthogonal protecting

groups at the side chains, TBTU (2 equiv.), HOBt (2 equiv.), DIPEA (4 equiv.), were added to the resin and the mixture was allowed to react for 10 min under MW irradiation (50 W, keeping internal temperature at 50 °C). The resin was washed 3 times with DMF (5 mL), MeOH (5 mL), DCM (5 mL). Coupling efficacy was monitored using the Kaiser test. Fmoc deprotection was carried out by treatment with 20% piperidine/DMF (5 mL) for 2 min under MW irradiation (40 W). The procedure was repeated, and after that, the resin was washed 3 times in sequence with DMF (5 mL), MeOH (5 mL), DCM (5 mL). For peptide cleavage, the peptidyl resin was treated with AcOH/TFE/DCM (1:1:4 v/v/v, 15 mL) under stirring for 90 min at rt. The resulting mixture was filtered, and the resin was washed in sequence with 10% TFA in Et_2O (5 ml), CH_2Cl_2 (5 ml) and MeOH (5 ml). The filtrate and the washes were collected, and the organic solvents were removed under reduced pressure. The resulting residue was suspended in ice-cold Et_2O, and the crude solid that precipitated was collected by centrifugation. The product was obtained as a waxy solid; 78% yield, 80% pure according to analytical RP HPLC (Method A, general methods), Rt = 6.71 min. MS (ESI): m/z calc. 1023.47, found: 512.8 $[M + 2H]^{2+}$, 1024.4 $[M + H]^{+}$.

Synthesis of c[Arg(Mtr)–Gly–Asp(OtBu)–D-Phe–Lys(Cbz)]

The crude linear peptide was dissolved in DMF (10 mL) and submitted to cyclization in the presence of $NaHCO_3$ (15 equiv.) under pseudo high-dilution conditions, by adding a solution of DPPA (3 equiv.) in DMF (10 mL) over 12 h using a temporized syringe, and the mixture was stirred for additional 6 h. Then the solvent was distilled under reduced pressure, and the residue was suspended in water (5 mL), and extracted 3 times with EtOAc (20 mL). The solvent was removed at reduced pressure, and the cyclopeptide was utilized without further purification; 67% yield, 75% pure according to analytical RP HPLC (Method B, general methods), Rt = 9.68 min. MS (ESI): m/z calcd. 1005.46; found: 1006.2 $[M + H]^{+}$, 1028.2 $[M + Na]^{+}$.

Synthesis of c[Arg(Mtr)–Gly–Asp(OtBu)–D-Phe–Lys(alkyne)]

The Cbz protecting group at Lys of c[Arg(Mtr)–Gly–Asp(OtBu)–D-Phe–Lys(Cbz)] was removed by catalytic hydrogenation over Pd/C in MeOH (20 mL) at rt for 3 h. After filtration of the catalyst over celite®, the solvent was distilled at reduced pressure.

The εNH_2 amine of Lys was derivatized with 5-hexynoic acid (1.2 equiv) in 1:2 DMF/DCM (15 mL) in the presence of HOBt/TBTU/DIPEA (2:2:4 equiv.) under MW (50 W, internal temperature 50 °C) for 15 min. The organic solvent was removed under reduced pressure, the residue was diluted with EtOAc (50 mL), and the organic phase was washed with 1M HCl (7 mL) and saturated $NaHCO_3$ (7 mL). The organic solvent was removed under reduced pressure, and the product was isolated as a waxy solid; 98% yield, 68% pure by analytical RP HPLC (Method B, general methods), Rt = 8.5 min. MS (ESI): m/z calc. 965.47; found: 966.4 $[M + H]^{+}$.

Synthesis of c[Arg–Gly–Asp–D-Phe–Lys(alkyne)] (cRGD–alkyne)

The final peptide side-chain deprotection was carried out by treatment of c[Arg(Mtr)–Gly–Asp(OtBu)–D-Phe–Lys(alkyne)] with TFA/thioanisole/phenol/triisopropylsilane (7:1:1:1 v/v, 10 mL) at rt for 8 h. Then the organic solvent was removed under reduced pressure, giving a white solid. The cyclopeptide–alkyne was isolated by preparative RP HPLC (general methods); 85% yield, 94% pure by analytical RP HPLC (Method A, general methods), Rt = 1.73 min. MS (ESI): m/z calc. 697.35; found: 349.6 $[M + 2H]^{2+}$, 698.2 $[M + H]^{+}$.

Synthesis of Boc–Ala–Val–Pro–Ile–Gly–OH

The linear Boc-protected pentapeptide was synthesized by SPPS on an H–Gly-preloaded 2-chlorotrityl resin according to the general procedure described above, using Boc–Ala–OH for the final coupling reaction. The chloranil test was utilized for monitoring the coupling between Fmoc–Val–OH and H–Pro–Ile–Gly–resin. Final peptide cleavage from the resin support with AcOH/TFE/DCM as described above, gave a white solid; 73% yield, 85% pure by analytical RP

HPLC (Method A, general methods), Rt = 5.22 min. MS (ESI): m/z calc. 555.33; found: 556.2 $[M + H]^+$.

Synthesis of Boc–Ala–Val–Pro–Ile–Gly–pent-4-yn-1-amine

The peptide Boc–Ala–Val–Pro–Ile–Gly–OH was solubilized in 1:2 DMF/DCM (15 mL), and HOBt/TBTU/DIPEA (2:2:4 equiv.) and 4-pentyn-1-amine (1.2 equiv.) were added to the solution. The reaction was carried out under MW radiation for 15 min (50 W, internal temperature 50 °C). EtOAc (50 mL) was added, and the organic phase was washed with 1M HCl (5 mL) and saturated $NaHCO_3$ (5 mL). The organic phase was removed under reduced pressure, and the product was obtained as yellowish oil; 75% yield, 80% pure by analytical RP HPLC (Method B, general methods), Rt = 7.24 min. MS (ESI): m/z calc. 620.39; found: 621.2 $[M + H]^+$, 643.2 $[M + Na]^+$.

Dansyl–AVPI–alkyne

Dansyl–Ala–Val–Pro–Ile–Gly–pent-4-yn-1-amine was obtained by the same procedure, using the commercially available dansyl-Ala-OH.

H–Ala–Val–Pro–Ile–Gly–pent-4-yn-1-amine (AVPI–alkyne)

The peptide Boc–AVPI–alkyne was treated with TFA/DCM (1:3 v/v, 4 mL) at rt for 25 min. The procedure was repeated, then the organic solvents were removed under reduced pressure. The product was isolated by preparative RP HPLC (general methods); 78% yield, 93% pure according to analytical RP HPLC (Method A, General methods), Rt = 2.3 min. MS (ESI): m/z calc. 520.34; found: 521.2 $[M + H]^+$, 543.2 $[M + Na]^+$.

Dimesylate derivative of Pluronic®F127

Before use, BASF Pluronic®F127 (PF127) surfactant (12.6 g, 1.0 mol, 1.0 eq.) was dried by azeotropic distillation at low pressure in toluene and was solubilized in anhydrous DCM (50 mL). This solution was cooled to 0 °C and kept under an inert N_2 atmosphere; after that, triethylamine (280 μL, 2.0 mmol, 2.0 eq.) and methanesulfonyl chloride (155 μL, 2.0 mmol, 2.0 eq.) were added. The reaction mixture was kept under stirring at 0 °C under an inert atmosphere for 3 h and then at rt overnight. The dispersion was filtered, the organic layer was distilled under reduced pressure, and the residue was dried under high vac. (quantitative yield).[1]

Diazide derivative of PF127, PF127–$(N_3)_2$

To a suspension of dimesylate–PF127 (10.2 g, 0.8 mmol, 1.0 eq.) in CH_3CN (50 mL), sodium azide (156 mg, 2.4 mmol, 4.0 equiv.) was added, and the reaction mixture was allowed to stir under reflux for 48 h. Subsequently, the solvent was removed under reduced pressure. The obtained solid was dispersed in 5% $NaHCO_3$ (10 mL), and the solution was then saturated with solid NaCl. This mixture was extracted four times with DCM (4 × 10 mL). The combined organic phases were dried over Na_2SO_4, filtered, and evaporated at reduced pressure, affording a white solid (>95% yield).

PEG-shell/silica-core NPs

PF127 (200 mg), PF127–$(N_3)_2$ (20 mg), and RhB-TES C (4.0 mg) were solubilized in DCM (2 mL) in a 20 mL glass scintillation vial. The organic solvent was evaporated from the homogeneous solution, and the solid residue was subsequently dried under vacuum at rt. The resulting solid mixture was solubilized at 25 °C while stirring in a 1 M AcOH/0.85 M NaCl solution (3.2 mL) for 1h. Subsequently, TEOS (350 μL) was added after 3h of stirring, then TMSCl (40 μL) was added. The mixture was kept under stirring for 48 h at 25 °C. The NPs were purified by dialysis against water at rt using cellulose dialysis tubing (Sigma, MW cutoff >12 kDa, avg. diameter 33 mm), and the solution was finally diluted to a total volume of 3.5 mL with water to the final NPs concentration 29 μM.[Error! Bookmark not defined.]

Peptide conjugation to NPs by CuAAC

A fragment of copper wire (1 cm) was polished in 10% HNO_3 for 2 min, rinsed with water and acetone and dried, and then introduced in a glass vial containing a dispersion of NPs (500 µL, 29 µM in water) in Tris buffer (1.5 mL, 200 mM in bidist. H_2O, pH 8). Subsequently, a $CuSO_4$ solution (6 µL, 2 mM in bidist. H_2O), sodium 4,4′-(1,10-phenanthroline-4,7-diyl)dibenzenesulfonate (12 µL, 2 mM in bidist. H_2O) and the peptide–alkyne (50 µL, 53 mM in DMSO) were added. The obtained homogeneous mixture was stirred for 3 days at rt. The NPs were purified via size-exclusion chromatography (SEC) on Sephadex® G-25 gel (dry bead size 20–80 µm, fractionation range 1000–5000 Da) using bidist. water as the eluent and finally diluted with bidist. water to obtain a final NP concentration of 3 µM. The NPs were stored at 4 °C.

Table S1. Summary of the hydrodynamic diameter data (water, 25 °C) for the samples presented in this work.

Sample	δH (by volume) / nm	δH (standard deviation) / nm	Polydispersion Index (PDI)
NP–N_3	22	0.5	0.10
AVPI–NPs	28	2	0.38
cRGD–NPs	29	4	0.29
AVPI/cRGD–NPs	28	2	0.40

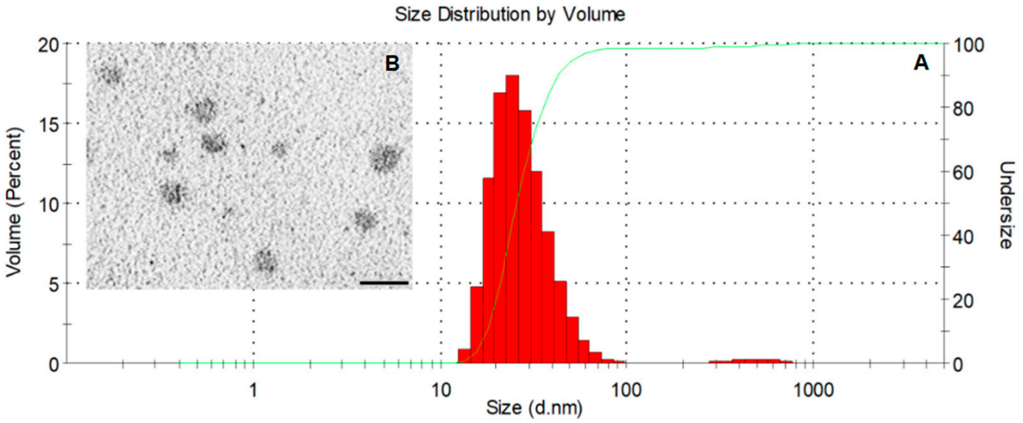

Figure S1. (**A**) DLS hydrodynamic size distribution by volume (water, 25 °C) and (**B**) representative TEM image of AVPI–NPs (scale bar 20 nm).

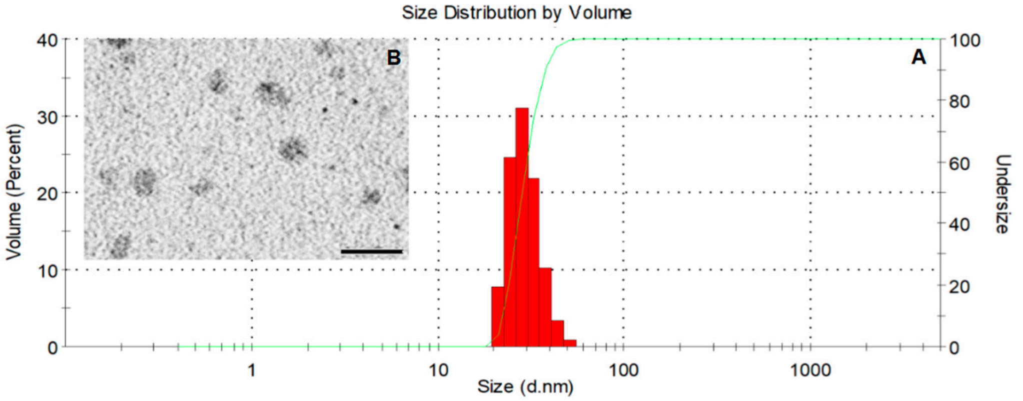

Figure S2. (**A**) DLS hydrodynamic size distribution by volume (water, 25 °C) and (**B**) representative TEM image of cRGD–NPs (scale bar 20 nm).

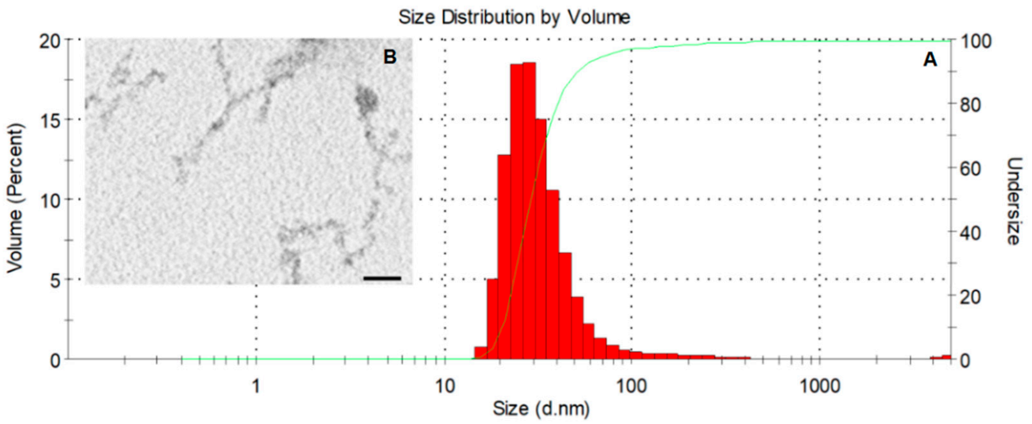

Figure S3. (**A**) DLS hydrodynamic size distribution by volume (water, 25 °C) and (**B**) representative TEM image of AVPI/cRGD–NPs (scale bar 20 nm).

Determination of NPs peptide functionalization

For fluorimetric quantitative analysis of peptides' primary amines on the NP surface, a PEG–amine (750 Da) standard calibration curve was established. In total, 2.5 mL DMSO and 500 µL of fluorescamine solution (0.264 mM in DMSO) were introduced in a UV-Vvis glass cuvette. Then, the fluorescence measurements with individual different quantities of PEG–diamine stock solution were conducted. The PEG–amine stock solution (0.675 mM in DMSO) was added at each measurement to obtain the following concentrations in the cuvette: 5.94 µM, 3.76 µM, 1.78 µM. After 8 min from the PEG–amine stock solution addition, the fluorescent signal was recorded (λex 390 nm, λem 480 nm). To quantify peptide functionalization, a sample of AVPI–NP conjugate (25 µL, 30 µM) was analyzed in the cuvette. According to the sample measurement, the number of 7.8 ± 1 peptides per NP was calculated.

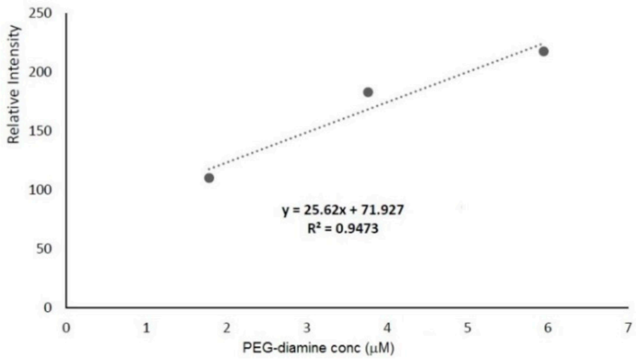

Figure S4. Calibration curve of the fluorescence intensity at λ 480 nm (λex 390 nm) vs. the concentration of PEG–NH2/fluorescamine standard solutions, constructed to measure the number of peptides/NP by titration with fluorescamine.

Quantification of dansyl–AVPI–NP

The NP–N_3 (29 mM, 500 µL) were reacted with dansyl–AVPI–alkyne or with a 1:1 mixture of dansyl–AVPI–alkyne and cRGD–alkyne, by CuAAC as described above. In short, NP–N_3 (500 µL, 29 µM) was diluted with Tris buffer (1.5 mL, 200 mM), and $CuSO_4$ aqueous solution (6 µL, 2 mM), sodium 4,4′-(1,10-phenanthroline-4,7-diyl)dibenzenesulfonate (12 µL, 2 mM), dansyl–AVPI–alkyne (50 µL, 53 mM in DMF), and polished copper wire were added under an inert atmosphere, and the mixture (final volume 2106 µL) was stirred for 48 h. The suspension was filtered with a 200 nm filter, and 400 µL of the filtrate was diluted to 5000 µL before purification by SEC using phosphate buffer (10 mM, pH 7.4), containing 1 mM EDTA. After purification, the concentration of dansyl–

AVPI–NP in solution was determined directly by fluorometric quantification against a calibration curve obtained by measuring the fluorescence intensity of standard solutions of dansyl–AVPI in the concentration range 1–9 mM (λex 340 nm, λem 477 nm). The calculation indicated a number of 9.3 ± 1 dansyl–AVPI peptides per NP. CuAAC functionalization of the NP–N3 (500 μL, 29 μM) was repeated with a 1:1 mixture of dansyl–AVPI–alkyne (12.8 μL, 53 mM in DMF) and cRGD–alkyne (12.8 μL, 53 mM in DMF). Fluorometric quantification indicated a number of dansyl–AVPI peptide/NP of 4.8. This number is suggestive of a circa 1:1 dansyl–AVPI/cRGD ratio, consistent with a very similar chemical reactivity for dansyl–AVPI–alkyne and cRGB–alkyne during CuAAC coupling.

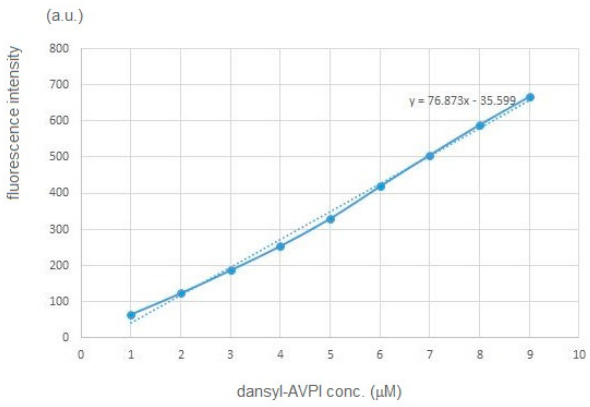

Figure S5. Calibration curve of the fluorescence intensity at λ 477 nm (λex 340 nm) vs. the concentration of dansyl–AVPI peptide standard solutions, constructed to determine the number of dansyl–AVPI/NP.

© 2020 by the authors. Submitted for possible open access publication under the terms and conditions of the Creative Commons Attribution (CC BY) license (http://creativecommons.org/licenses/by/4.0/).
